# Simultaneous Heterotrophic Nitrification and Aerobic Denitrification of Water after Sludge Dewatering in Two Sequential Moving Bed Biofilm Reactors (MBBR)

**DOI:** 10.3390/ijerph19031841

**Published:** 2022-02-06

**Authors:** Eshetu Janka, Sabin Pathak, Alireza Rasti, Sandeep Gyawali, Shuai Wang

**Affiliations:** 1 Department of Process, Energy and Environmental Technology, University of South-Eastern Norway, 3918 Porsgrunn, Norway; spathak702@gmail.com (S.P.); 238780@student.usn.no (A.R.); sandeep.gyawali@usn.no (S.G.); 2 Biowater Technology AS, 3115 Tønsberg, Norway; sw@biowatertechnology.com

**Keywords:** biofilm, reject water, carriers, sequencing, heterotrophic

## Abstract

Water after sludge dewatering, also known as reject water from anaerobic digestion, is recycled back to the main wastewater treatment inlet in the wastewater treatment plant Porsgrunn, Norway, causing periodic process disturbance due to high ammonium of 568 (±76.7) mg/L and total chemical oxygen demand (tCOD) of 2825 (±526) mg/L. The main aim of this study was the simultaneous treatment of reject water ammonium and COD using two pilot-scale sequential moving bed biofilm reactors (MBBR) implemented in the main wastewater treatment stream. The two pilot MBBRs each had a working volume of 67.4 L. The biofilm carriers used had a protected surface area of 650 m^2^/m^3^ with a 60% filling ratio. The results indicate that the combined ammonia removal efficiency (ARE) in both reactors was 65.9%, while the nitrite accumulation rate (NAR) and nitrate production rate (NPR) were 80.2 and 19.8%, respectively. Over 28% of the reject water’s tCOD was removed in both reactors. The heterotrophic nitrification and oxygen tolerant aerobic denitrification were the key biological mechanisms found for the ammonium removal in both reactors. The dominant bacterial family in both reactors was *Alcaligenaceae*, capable of simultaneous heterotrophic nitrification and denitrification. Moreover, microbial families that were found with equal potential for application of simultaneous heterotrophic nitrification and aerobic denitrification including *Cloacamonaceae*, *Alcaligenaceae*, *Comamonadaceae*, *Microbacteriaceae*, and *Anaerolinaceae*.

## 1. Introduction

In conventional wastewater treatment processes, the reject water, which is the water after sludge dewatering from anaerobic digestion effluent, is directly recycled into the main inlet without any pre-treatment. Normally, reject water that is recycled to the inlet is about 1–2% of the main flow. However, reject water is highly concentrated wastewater that contains up to 25% of the total nitrogen load of the mainstream [1]. The main reject water constituents are ammonium (ca. 600 mg/L) as well as slowly degradable chemical oxygen demand (COD) (ca. 2 to 3 g/L). Mostly, the COD in reject water is associated with a low fraction of biodegradable substances. Occasionally, the high ammonium and COD concentration in reject water may cause process disturbance when recycled in the main treatment system. Hence, to avoid overload and process disturbance in the main treatment process, the biological treatment of reject water is vital [2].

The conventional biological treatment processes for the treatment of nitrogen in reject water involved mainly nitrification by autotrophs under aerobic conditions and denitrification by heterotrophs under anaerobic conditions [3,4,5]. Nitrification is a biological process that takes place by the two autotrophic bacteria called ammonia-oxidizing bacteria (AOB) and nitrite-oxidizing bacteria (NOB) [4,6]. However, the typical AOB and NOB bacteria species known as *Nitrosomonas* and *Nitrobacter*, respectively, may not grow well or survive in wastewater containing high free ammonia as well as other toxic compounds [6,7,8]. For instance, the inhibitory effects of reject water on NOB were due to increased concentrations of free ammonia [9]. Furthermore, industrial effluents contain high concentrations of toxic compounds such as phenols, cyanides, and thiocyanate inhibit the AOB and NOB activities [8].

It has been found that under such adverse environmental conditions for autotrophic bacteria, nitrification, and denitrification could also occur with the help of heterotrophic nitrifying bacteria as well as oxygen tolerant denitrifying bacteria [4,6,10,11]. It has been reported that there are several potential bacteria species capable of combined heterotrophic nitrification and aerobic denitrification in biological nitrogen removal systems [3,12,13,14]. Besides the biochemical mechanisms of heterotrophic nitrification, the bacteria possess ammonia and hydroxylamin-oxidizing enzymes to oxidize NH_4_^+^ to NO_2_^−^ as well as a large number of heterotrophic microorganisms that have the ability to convert NO_2_^−^ to NO_3_^−^ [11,15,16]. The heterotrophic nitrification and aerobic denitrification, which occur simultaneously, have the following stoichiometric formula [6,17,18].

(1) $${\mathrm{NH}}_{4}\to \;{\mathrm{NH}}_{2\;}\mathrm{OH}\;\to \;{\mathrm{NO}}_{2\;}\to {\mathrm{NO}}_{3}$$

(2) $${\mathrm{NO}}_{3}\to {\mathrm{NO}}_{2-}\to \mathrm{NO}\to N_{2}O\to N_{2}$$

Additionally, it has been reported that some specific bacteria species have shown the ability to convert ammonium to nitrogen under aerobic via a hydroxylamine intermediate instead of nitrate and nitrite reductase activity [6,19].

The heterotrophic nitrification and aerobic denitrification have many potential advantages in wastewater treatment. These major advantages are (i) simultaneous nitrification and denitrification, (ii) fewer acclimation problems, and (iii) compensation of alkalinity (i.e., alkalinity consumption in nitrification will be compensated alkalinity generated during denitrification) [3,20].

Moving bed biofilm reactors (MBBR) have shown a promising result in treating reject water through nitrification and denitrification processes. The MBBR is suitable for simultaneous nitrification–denitrification because of oxygen diffusion through the biofilm and can maintain an aerobic environment inside and outside of biofilm as well as the growth of suspended biomass. However, the efficiency of MBBR depends on the type of carrier material and the percentage of carrier filling. In particular, to avoid detachment of biofilms, some novel carriers are being developed through physical and chemical surface modifications to enhance biofilm adhesion to the carriers [21]. Moreover, flow and mixing conditions are the crucial parameter to maintain appropriate turbulence, which maintains the suitable thickness of biofilm suitable for full-substrate penetration [18]. High turbulence causes more detachment of the biofilm from the carrier, and low turbulence results in slower movement of the carrier and higher thickness of microorganisms in the biofilm.

**Figure 1:**
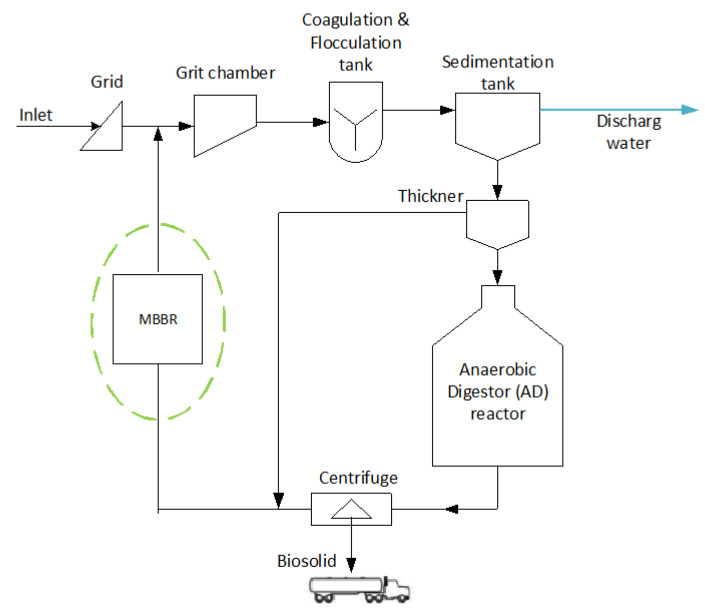
Sketch of the wastewater treatment plant and flow of reject water recycled to the main treatment stream after being treated by MBBR.

Few studies have been conducted by implementing a pilot case of sequential MBBR for reject water treatment using simultaneous heterotrophic nitrification and aerobic denitrification in the main wastewater treatment stream (Figure 1). Hence, this study has proposed implementing two sequential MBBR to treat reject water before it is recycled to the main inlet to improve the overall treatment efficiency of wastewater. The nitrogen and organic removal of the reactors were analyzed during the experimental period. Moreover, to promote the simultaneous heterotrophic nitrification and aerobic denitrification process, the development of the bacterial communities was identified through microbial population sequencing analysis. This study promotes the use of MBBR and simultaneous heterotrophic nitrification and aerobic denitrification process in reject water treatment for two reasons. Firstly, the slowly degradable particulate and colloidal organics in the reject water will be biodegraded. Secondly, the simultaneous heterotrophic nitrification and aerobic denitrification process reduce the nitrogen load due to the recycling of reject water to the main inlet where the treated reject water will cause less disturbance in the treatment process.

## 2. Materials and Methods

### 2.1. Experiment Setup

**Figure 2:**
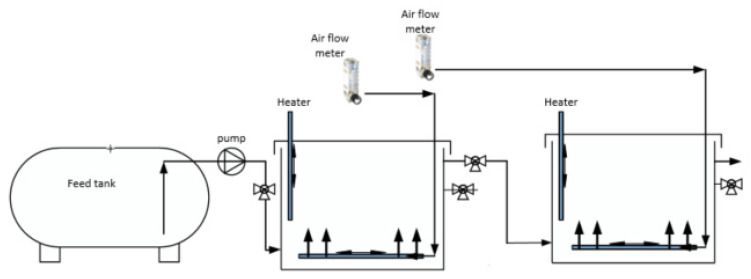
The two MBBR reactors setup at the treatment plant.

Two pilot moving bed biofilm reactors (MBBRs), from now onwards called MBBR R1 and MBBR R2, made of stainless steel and polycarbonate were set up in series for this experiment (Figure 2). Each MBBR has a length, breadth, and height of 0.35, 0.35, and 0.55 m, respectively, resulting in a total volume of 67.4 L. The biofilm carriers used were BTW S^®^ (Biowater Technology AS, Tønsberg, Norway) type with dimensions of 14.5 × 18.5 × 7.3 mm and a protected surface area of 650 m^2^/m^3^. The total protected area was calculated using the working volume of the reactors and the carrier filling, which was 60% in each reactor. The water temperature in both reactors was set at 30 (±2) °C. The water was heated by an aquarium heater (EHEIM 300 W, max 1000 L, Germany), and to avoid temperature loss to the surrounding the reactors were covered by black PVC/NBR rubber plastic insulation sheets. MBBR R1 was aerated continuously while the aeration in MBBR R2 was intermittent. The flow of aeration in both reactors was controlled and regulated by an air flow meter with an air flow rate set to 22 L/min. The detail design and operating parameters of pilot-scale sequential MBBR reactor within the experimental period is demonstrated in Table 1.

### 2.2. Reject Water Characteristics and Chemical Analysis

The feed for both reactors was reject water from the centrifuge of the anaerobic digester (Figure 1). The reject water coming directly from the centrifuge was stored in a 1 m^3^ HDPE plastic IBC tank and pumped to MBBR R1 using a Watson Marlow 520 s peristatic pump (WATSON MARLOW, Falmouth, the UK). The effluent of MBBR R1 is the inlet of MBBR R2. The setup of the pilot rectors is shown in Figure 2.

To analyze the water characteristics of the reject water three samples were collected twice a week. The three samples were the inlet to MBBR R1, outlet of MBBR R1, and outlet of MBBR R2. The samples were collected using a standard procedure and kept in a refrigerator before complete analysis of all the physical, inorganic, and organic chemical constituents in the reject water. The analyses include pH, total and soluble chemical oxygen demand (tCOD and sCOD), NH_4_-N, NO_2_-N, NO_3_-N, total solids (TS), total suspended solids (TSS), total volatile solids (TVS), volatile suspended solids (VSS), and alkalinity.

The tCOD and sCOD were measured by chemical wet oxidation in a closed glass vial by using a spectroquant^®^ pharo 300 UV/VIS photometer (Darmstadt, Germany). For the tCOD analysis, the samples were first homogenized by an overhead stirrer for 2 to 3 min. Two milliliters of the sample was pipetted into spectraquant COD cells in the measuring range of 300 mg/L to 3500 mg/L. To measure sCOD, the sample was centrifuged at 10,000 rpm for 30 min and then filtered at 0.45 µm (GxF multilayered, Acordisc^®^ PSF syringe filters) pore size before analysis. The COD method corresponds to US standard 5220 D [22]. For ammonium nitrogen (NH_4_-N) analysis, the samples were centrifuged. The samples were then diluted with milli-Q water by dilution factor 50×. A volume of 0.1 mL of the sample was pipetted into a spectraquant ammonium-nitrogen cell test with a measuring range of 4.0 mg/L to 80 mg/L. The method is analogous to US standard 4500-NH3 [22]. For the nitrite-nitrogen (NO_2_-N) and nitrate-nitrogen (NO_3_-N) analyses, samples were centrifuged and filtered before analysis. For alkalinity, concentration was measured as CaCO_3_ mg/L in the photometer at 605 nm. The pH of samples was measured by a Beckman 390 pH meter after calibration with buffer solutions of pH 4.0 and 7.0. Temperature and dissolved oxygen (DO) were measured using a WTW Oxi 3310 (Weilheim, Germany) oxygen meter. Total suspended solids (TSS), volatile suspended solids (VSS), and pH were also measured according to the U.S. standard 2540 D, 2540 E, and 4500-H [22], respectively.

### 2.3. Reject Water Element Analysis

The reject water consists of different kinds of metal constituents. To identify these different constituents of metals the inductively coupled plasma-mass spectrometry (ICP-MS) method was applied. The samples were first diluted in 5% HNO_3_ and analyzed on Agilent 8800 Triple Quadropole ICP-MS (ICP-QQQ) with an SPS 4 Autosampler. The analysis results are quantified against certified reference materials (CR) and inorganic internal standards.

### 2.4. Biomass Growth on Carriers

The biomass growth was per unit protected surface area (g/m^2^) was calculated from the measurement of total suspended solids (TSS) in g/L per surface area of carriers 650 m^2^/m^3^. The biomass on carriers was measured using ten carriers sampled out every week from each reactor. The carriers were dried at 105 °C for 24 h. The dried and cooled carriers were weighed as first weight (*m*_1_). After the first weight was measured, the carriers were washed with sodium hypochlorite solution (NaOHCl) and tap water thoroughly to remove the attached biomass. After cleaning the biomass, the carriers were let to dry at 105 °C for 24 h. The dried and carriers were weighed as second weight (*m*_2_). Biomass per carrier was calculated as:

(3) $$Biomass\;per\;carrier\;\left(m\right)=\frac{m_{1}-m_{2}}{N}$$

(4) $$W=m\times \left[\frac{VC}{A}\right]$$

where *W* is biomass per unit protected surface area (g/m^2^), m is biomass per carrier, *VC* is the number of pieces per m^3^ carrier, and *A* is the protected surface area (m^2^/m^3^).

The specific nitritation rate (SNR), which is the nitrite produced per total surface area of carrier per day (mg NO_2_-N/m^2^·d) and specific denitrification rate (SDR), which is the total ammonium reduced per total surface area of carrier per day (mg N_2_/m^2^·d) in MBBR R1 and MBBR R2 were calculated as follows:

SNR = ([NO_2_^−^ − N]eff.) − ([NO_2_^−^ − N]inf.) × Q])/A (5)

SDR = ([NH_4_^+^ − N]inf. + [NO_2_^−^ − N]inf. + [NO_3_^−^ − N]inf.) − ([NH_4_^+^ − N]eff. + [NO_2_^−^ − N]eff. + [NO_3_^−^ − N]eff.) × Q])/A (6)

where Q is the flow (L/day) and A is the total surface area of the biocarrier (m^2^). The ammonia removal efficiency (ARE) and the nitrite accumulation rate (NAR) were calculated as:

ARE = ([NH_4_^+^ − N]inf. − [NH_4_^+^ − N]eff.)/([NH_4_^+^ − N]inf.) × 100 (7)

NAR = ([NO_2_^−^ − N]eff.)/([NO_2_^−^ − N]eff. + [NO_3_^−^ − N]eff.) × 100 (8)

### 2.5. Microbial Analysis

#### 2.5.1. Microbiome DNA Extraction

For the microbiome genomics analysis, samples from both reactors were collected according to the DNA extraction procedure where samples were collected in such a way that no cross-contamination occurred during sampling. The samples were stored in a cold refrigerator in a separate kit to avoid any further cross-contamination before the genomic analysis. The standard protocol for DNA extraction and amplification from soil samples that produces DNA pure enough for PCR amplification was applied [23].

The samples were first well mixed and transferred to separate 30 mL test tubes. Meanwhile, the remaining samples were stored as glycerol stocks and stored at −80 °C as reserve stock. The 30 mL samples were centrifuged at 4000 rpm (revolution per minute) for 10 min, at 4 °C. The supernatants were transferred to new tubes for chemical analysis, while precipitate cell mass was used for making pellets. The cell mass was taken from the pellet for DNA extraction using a Fast DNA SPIN Kit for soil. Whenever needed the cell mass was re-suspend in extraction buffer. DNA extracted from all samples with sufficient yield and purity for further 16S analysis.

#### 2.5.2. Microbiome Gene Amplification by Polymerase Chain Reaction (PCR)

The DNA was amplified by polymerase chain reaction (PCR) and the targeted regions of the bacterial 16S rRNA gene were amplified using primers. Amplicon PCR of 16S fragment amplified in all samples as well as an image of agarose gel (600 bp) was produced showing amplified 16S fragment of all samples. The recovered PCR product for sequencing was carried out using illumina Miqeq sequencing (i.e., 16S metabarcoding and microbial annotation). The sequence was illustrated into different operational taxonomic units (OUT) based on the similarity [24].

### 2.6. Data Analysis

The data generated from the biochemical analysis and onsite measurements were processed in Microsoft Excel for data visualization, mass balance analysis, and standard plotting. The mean of each biochemical and physical parameter measurement over time was used for the statistical comparisons between the different measurement periods.

## 3. Results

### 3.1. Ammonium Transformation in MBBR Reactors

**Figure 3:**
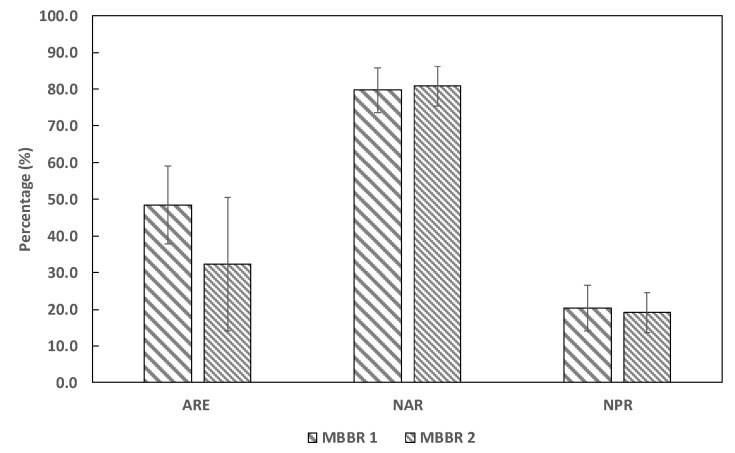
The ammonium removal efficiency (ARE), nitrite accumulation rate (NAR), and nitrate production rate (NPR) in both reactors (*n* = 23).

The average inlet reject water ammonium concentration fed to MBBR R1 during the experimental period was 568 (±76.7) mg/L. Therefore, this resulted in an average of 51.1 (±6.9) mg/m^2^·d specific ammonium loading rate (SALR) per square meter of carrier surface. In the biological treatment process, the ammonia in MBBR R1 was close to half converted to nitrite, and the effluent ammonia was reduced to 296.3 (±81.7) mg/L. Hence, in MBBR R1 the ammonia removal efficiency (ARE) was 48.8%, while the nitrite accumulation rate (NAR) and nitrate production rate (NPR) were 79.7% and 20.3%, respectively (Figure 3). The effluent of the MBBR R1 fed as an inlet for MBBR R2 had a SALR of 25.8 (±7.3) mg/m^2^·d. Likewise, the ARE, NAR, and NPR in MBBR R2 was 32.3, 80.8, and 19.2%, respectively.

In terms of specific nitritation rate per carrier surface area per day, (SNR) MBBR R1 was 66% higher than MBBR R2 (Table 2). Whereas the specific denitrification rate per carrier surface area in MBBR R2 was 25% higher than in MBBR R1. This shows that there was relatively higher nitritation in MBBR R1 while the relative denitrification was higher at MBBR R2.

### 3.2. Chemical Oxygen Demand (COD)

**Figure 4:**
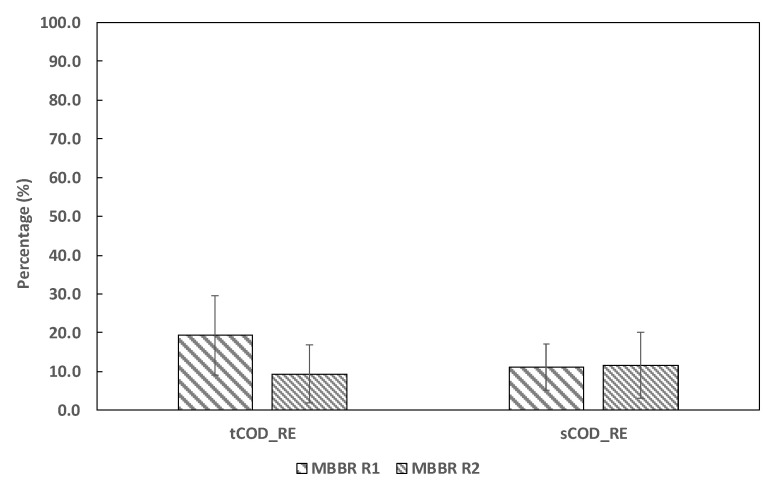
The total and soluble removal efficiency (tCOD_RE and sCOD_RE) in MBBR R1 and MBBR R2 reactors (*n* = 23).

The inlet reject water fed to MBBR R1 had on average total COD (tCOD) and soluble COD (sCOD) of 2825 (±526) mg/L and 1978 (±314) mg/L, respectively. The tCOD and sCOD removal efficiency (tCOD_RE and sCOD_RE) in MBBR R1 were 19.3 (±10.2) and 11.1 (±6.0)%, respectively (Figure 4). Since the effluent of MBBR R1 was fed to MBBR R2, the remaining slowly degradable COD was consumed and the tCOD and sCOD removal efficiency in MBBR R2 were 9.3 (±7.7) and 11.6 (±8.5)%, respectively. When combined, both reactors removed 28.8 (±9.2) and 17.7 (±9.2) percent of tCOD and sCOD of the inlet reject water, respectively.

### 3.3. The Reject Water Element Composition

**Figure 5:**
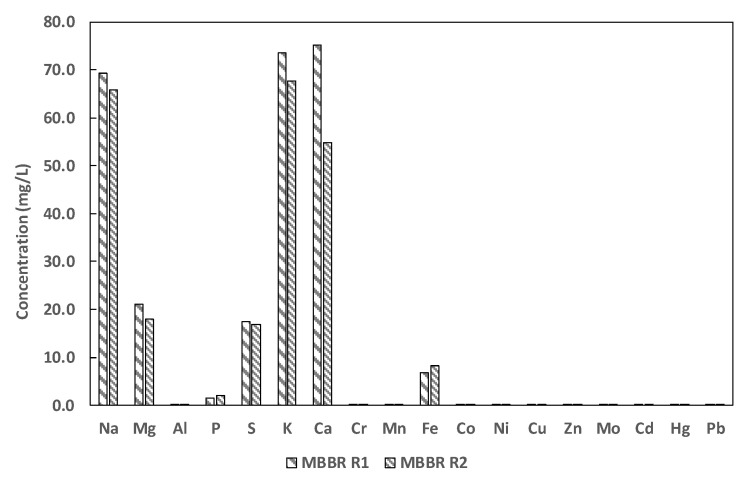
The major metallic and non-metallic element concentrations in the reject water from MBBR R1 and MBBR R2.

The metallic and nonmetallic element analysis of the reject water both from the effluents of the MBBR R1 and MBBR R2 is shown in Figure 5. There was a slight difference in the concentration of the metallic and non-metallic elements found in both reactors. However, in both reactors calcium (Ca), potassium (K), sodium (Na), magnesium (Mg), and iron (Fe) are the major metallic elements in the reject water in the order mentioned. To a lesser extent, none metallic elements such as sulfur (S) and phosphorous (P) were also found.

### 3.4. Biomass on the Carriers

The biomass growths on the carriers in reactors MBBR R1 and MBBR R2 were in the range of 74–128 g/m^2^ and 48–128 g/m^2^, respectively. There was a large variation in biomass accumulation in both reactors over time. However, the carriers in MBBR R1 accumulated larger biomass on average than the carriers in MBBR R2. Therefore, with similar carriers having equal total surface areas, in most of the cases, the biofilm concentration was higher in MBBR R1 than in MBBR R2.

### 3.5. Microbial Community in the Reactors

**Figure 6:**
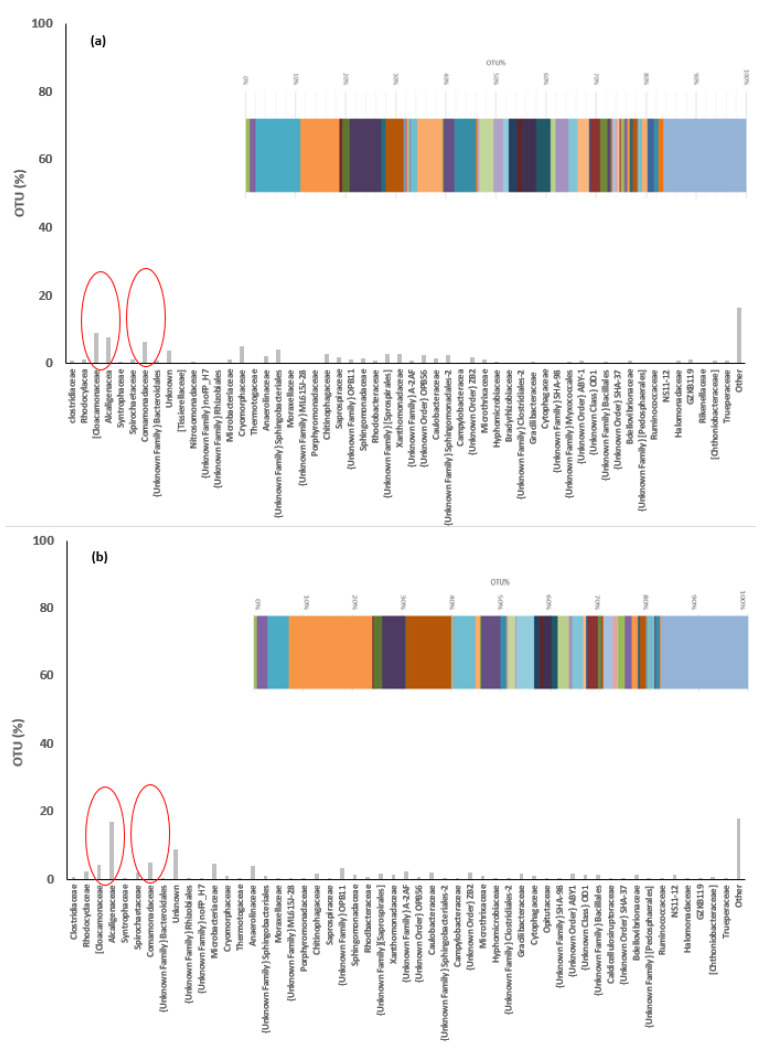
The operational taxonomic units (OUT) and taxonomic identities of bacterial biomass in the sequencing at the family level in MBBR R1 (**a**) and MBBR R2 (**b**) reactors.

The overall microbial community composition in both MBBR R1 and MBBB R2 is clustered into different operational taxonomic units (OUT) as shown in Figure 6. There was a very diverse bacterial community and a slight difference in bacterial species abundance and richness between the two reactors. The major dominant microbial families in MBBR R1 were *Cloacamonaceae*, *Alcaligenaceae*, and *Comamonadaceae*. While in MBBR R2 the most dominant microbial families were *Alcaligenaceae* but the species *Cloacamonaceae* and *Comamonadaceae* were also present in addition to other microbial families such as *Microbacteriaceae* and *Anaerolinaceae*. However, there was also a substantial proportion of the OUT that showed an unknown bacterial population.

## 4. Discussion

### 4.1. Ammonium Transformation to Nitrite and Nitrate

The ammonium concentration in both reactors (i.e., MBBR R1 and MBBR R2) transformed largely to nitrite but also to nitrate and nitrogen gas too (Figure 3). It was reported that the nitrogen removal in MBBR reactors depends mainly on the types of microbial community and the functional features of the bacterial population. Autotrophic nitrification is the most common nitrification process in wastewater treatment by chemolithoautotrophic AOB and NOB bacteria communities [11,21,25]. The most recognized autotrophic AOB genus is *Nitrosomonas* in the family of *Nitrosomonadaceae* whereas the NOB genus is *Nitrobacter* in the family of *Nitrobacteraceae*. In our study, the operational taxonomic units (OUT) and taxonomic identities of bacterial biomass in the sequencing analysis in both reactors showed that none of these autotrophic nitrifier genera were found in both reactors (Figure 6. Therefore, we concluded that the AOB and NOB were completely inhibited due to the high concentration of free ammonia or other unknown toxic constituents in the system. It was reported that the AOB and NOB bacteria species might not survive in wastewater containing high free ammonia as well as other toxic compounds [6,7,8,9]. A free ammonia (FA) concentration higher than 8–120 mg/L inhibits the AOB bacteria, while 0.08–0.82 mg/L FA concentrations hinder NOB activity [26]. For instance, free ammonia (FA) as low as 0.6 mg/L inhibits NOB [21]. In this study, the FA was much higher in both reactors, 14.9 (±13.6) mg/L and 2.9 (±4.4) mg/L in MBBR R1 and MBBR R2, respectively. On top of that, the reject water may contain some toxic compounds that may have inhibited the AOB and NOB activities [8].

Therefore, the most plausible nitrification process that happened in both reactors was heterotrophic nitrification and aerobic denitrification. The types of microbial communities found in the sequencing analysis supported this conclusion. The microbial community analysis showed that the higher out% and the dominant microbial families in MBBR R1 were *Cloacomonaseae*, *Alcaligenaceae*, *Comamonadaceae*, and *Cryomorphaceae*. Similarly, the dominant microbial community in the MBBR R2 were *Alcaligenaceae*, *Comamonadaceae*, *Microbacteriaceae*, *Cloacomonaseae*, *Anaerolinaceae*, and others (Figure 6).

The heterotrophic aerobic ammonia oxidation known as heterotrophic nitrification is one of the biological nitrogen removal processes carried out by a diverse and wide range of bacterial communities using organic substrates as energy sources to oxidize ammonia [11,25,27]. It is also reported in several studies that many heterotrophic nitrifying bacteria can also be capable of aerobic denitrification [25]. The dominant bacterial family *Alcaligenaceae* found in both reactors are capable of simultaneous nitrification and denitrification. In a selective enrichment of *Alcaligenaceae*, Kalniņš [6] used this family from industrial wastewater for simultaneous nitrification and denitrification of wastewater. Four strains representing the *Alcaligenaceae* family have been isolated from the green water system for their ability to nitrify ammonia and nitrite aerobically [10].

The reject water has sufficient soluble and slowly degradable COD, which was used by these groups of bacteria (Figure 4). Hence, heterotrophic nitrification has an advantage through simultaneous organic removal especially for reject water that consists of a large fraction of degradable organics.

### 4.2. Aerobic Bacterial Denitrification

The most common denitrification is the conversion of nitrates to nitrogen gas by the facultative chemo-organoheterotrophic bacterial communities under anoxic conditions [11,25,28]. In our experiment, both reactors were well aerated (Table 1), but there was sufficient denitrification in both reactors (Table 2). There could exist some anoxic microenvironment inside the biofilm on carriers [21,29]. However, the microbial community analysis showed the existence of an oxygen-tolerant aerobic denitrifying bacterial community. Many heterotrophic nitrifiers such as the *Alcaligenaceae* family can also carry out aerobic denitrification. Aerobic denitrifiers tend to work efficiently at 25–37 °C and pH 7–8 when dissolved oxygen concentration is 3–5 mg/L and the C/N load ratio is 5–10 [30]. The most extensively characterized aerobic denitrifying bacterium, *Paracoccus denitrificans* reduced 27% of added nitrate to gaseous nitrogen in the presence of oxygen [11,25,31]. In both reactors, the bacterium in the families of *Alcaligenaceae, Comamonadaceae, Microbacteriaceae, Cloacomonaseae,* and *Anaerolinaceae* can carry out denitrification in the presence of oxygen or in any anoxic microenvironment created on the carriers. Most of the denitrifiers reported in solid-phase denitrification are affiliated to the family *Comamonadaceae* [32]. Moreover, several of the families correlated with the denitrification rates were significantly associated with the families such as *Anaerolinaceae* and *Microbacteriaceae*, suggesting that these families potentially play an important role in denitrification [33]. The *Cloacomonaseae* family of bacterial communities is common in anaerobic digesters as a denitrifier [34].

Aerobic denitrification is a good alternative to conventional denitrification for its unique advantage of allowing simultaneous nitrification and denitrification in one aerated reactor [30,31]. Moreover, for reject water that consists of a high C/N ratio, a combination of nitrification and denitrification in one aerated reactor has the advantage of simultaneous organic removal.

### 4.3. Biomass Growth and Metallic Metallic Elements

The dynamics of biomass concentration on the carriers in both reactors were in the range of 48–128 g/m^2^ during the operational period. The biomass accumulation in both reactors had a large variation and did not stabilize over time. Several conditions such as carrier type (i.e., size, shape, and specific surface area), filling ratio, hydrophilicity, and electrophilicity of bio-carriers affect biofilm growth and stable accumulation on the carriers [21,35]. Moreover, some nitrifying bacteria form thin biofilm on the carriers due to their poor EPS (extracellular polymerase) production. However, the study showed that there is a possibility of enhancing nitrifying biofilm formation rate with the aid of EPS produced by heterotrophic bacteria [21,36].

In a study to improve the hydrophilicity and electrophilicity of carriers, iron oxide (Fe_2_O_3_) was used due to the positive electricity [21]. Iron affects biofilm formation in some other bacteria and especially ferrous (Fe^2+^) and ferric (Fe^3+^) iron-stimulated biofilm formation [37]. In this study, the reject water had a substantial amount of Fe (Figure 5) and this may help with the biomass formation. However, excessive biofilm accumulation and scaling on the bio-carriers should get due attention. Scaling on the biofilm carriers can occur when there is a high concentration of ammonium, phosphorus, and metal ions causing biofilm carriers to sink to the bottom of reactors. Scaling causes less carrier motion and requires higher energy consumption, causing lower process efficiency and increased operational cost. In this study the excessive Fe^3+^ and Ca^2+^ ions may have a potential to form mineral precipitates and scaling on the biofilm carriers [2,38].

## 5. Conclusions

The ammonium concentration in both reactors transformed largely to nitrite but also to nitrate and nitrogen gas too. The combined ammonia removal efficiency (ARE) in both reactors was 65.9%, while the nitrite accumulation rate (NAR) and nitrate production rate (NPR) were 80.2% and 19.8%, respectively. The heterotrophic aerobic ammonia oxidation known as heterotrophic nitrification and oxygen tolerant aerobic denitrifying were the identified biological mechanisms for the ammonia removal in both reactors. The dominant bacterial family *Alcaligenaceae* found in both reactors and other related bacterial species are capable of simultaneous nitrification and denitrification.

Moreover, the simultaneous heterotrophic nitrification and denitrification removed over 28% of the reject water’s tCOD combined in both reactors. Hence, simultaneous heterotrophic nitrification and aerobic denitrification have many potential advantages in reject water treatment. In addition to ammonia removal, heterotrophic nitrification and denitrification have additional advantages for organic removal especially for reject water that consists of a large fraction of slowly degradable organics because heterotrophic nitrifiers and aerobic denitrifiers use organic substrates as energy sources to oxidize ammonia.

The very diverse bacterial communities identified need a strategic and targeted enrichment of heterotrophic nitrification and aerobic denitrification for future application. In conclusion, the dominant microbial families that have the potential for application of simultaneous heterotrophic nitrification and aerobic denitrification are *Cloacamonaceae*, *Alcaligenaceae*, *Comamonadaceae, Microbacteriaceae*, and *Anaerolinaceae*.

## Figures and Tables

**Table 1:** The operating parameters used in the experiment in both reactors.

	Units	MBBR R1	MBBR R2
Reactor volume	L	67.4	67.4
Temperature	°C	30 (±2)	30 (±2)
Water depth	m	0.61	0.57
Type of media	--	BWT S^®^	BWT S^®^
Surface area of carriers	m^2^/m^3^	650	650
Total projection surface area	m^2^	26.3	26.3
Filling rate	%	60	60
HRT	day	1.2	1.2
COD loading	kg/m^3^·d	2.4 (±0.4)	1.9 (±0.2)
NH4-N loading	kg/m^3^·d	0.48 (±0.1)	0.25 (±0.1)
Dissolved oxygen (DO)	mg/L	3.8 (±2.4)	3.8 (±2.4)

**Table 2:** The specific nitritation and denitrification in MBBR R1 and MBBR R2.

Reactors	Specific Nitritation Rate (SNR) (mg NO_2_/m^2^·d)	Specific Denitrification Rate (SDR) (mg N_2_/m^2^·d)
MBBR R1	468.7 (±137.8)	55.0 (±101.8)
MBBR R2	157.3 (±105.1)	73.1 (±81.0)

## Data Availability

Not applicable.
